# Inclusive Leadership and Creative Territory Behavior: A Triple Interactive Moderating Effect Model

**DOI:** 10.3390/bs15081105

**Published:** 2025-08-14

**Authors:** Guanfeng Shi, Ziyi Zhang

**Affiliations:** 1 School of Economics and Management, Shihezi University, Shihezi 832003, China; 2 Department of Corporate Governance and Management Innovation Research Center, Shihezi University, Shihezi 832003, China

**Keywords:** inclusive leadership, creative territory behavior, harmonious work passion, work autonomy, status competitive motivation

## Abstract

Based on self-determination theory and the “environment–cognition–behavior” analysis framework, harmonious work passion is introduced into the research system to systematically explore the mechanism and internal path of inclusive leadership on employees’ creative territory behavior. Combined with work autonomy and status competition motivation, a three-way interaction model is constructed to reveal the boundary conditions under which inclusive leadership affects employees’ creative territory behavior. Through situational experiments (Study 1) and multi-time questionnaire surveys (Study 2), the results showed that harmonious work passion mediates the negative impact of inclusive leadership and creative territory behavior; when work autonomy is strong and employees’ status-competitive motivation is high, inclusive leadership has the most significant effect on creative territory behavior through harmonious work passion. The interaction among inclusive leadership, work autonomy, and status-competitive motivation is significant. The purpose of this study is to provide practical guidance for managers to reduce employees’ negative behaviors by optimizing the work environment and incentive strategies.

## 1. Introduction

Although idea generation is the starting point of innovative activity (Tang & Zhang, 2015), individual ideas can only achieve their value if they gain recognition and support through communication and interaction. However, due to the ambiguity of the property rights boundary of creative achievements, employees often protect core resources by hiding ideas and refusing to cooperate due to concerns about the loss of competitive advantage, which significantly inhibits the healthy development of organizational innovation. Creative territory behavior is the self-protection behavior of employees to protect creative achievements in order to safeguard their own rights and interests. It is noteworthy that existing studies have mainly explored the stimulating effect of negative leadership models (Huang & Tang, 2020), self-serving leadership, and paradoxical leadership (Cao & Zhao, 2022) on employees’ creative territory behavior (Huang & Tang, 2020; Tan & Yuan, 2024), while ignoring the possible inhibitory effect of inclusive leadership (Kong et al., 2020), which emphasizes openness and sharing. Inclusive leadership creates psychological security by accepting work mistakes and encouraging different opinions (Q. X. Zhu, 2011), which may be offset with defensive psychology such as in relation to the knowledge disclosure fear behind creative territory behavior. However, its specific mechanism and boundary conditions have not been systematically verified. Filling this theoretical gap has important practical significance for organizations to construct leadership intervention programs to inhibit territory behavior. Therefore, it is very important to motivate employees’ innovative behavior, eliminate the ice in their territory, and release their kinetic energy and vitality. Under the Chinese scenario of “all rivers are accepted in the sea, tolerance is great”, organizations often emphasize collective harmony and hierarchical relations. Organizations also urgently need leaders to develop inclusive leadership practices that are characterized by understanding the differentiated needs of employees and accommodating their individual characteristics to enhance the organization’s innovation capabilities and core competitiveness (Tang & Zhang, 2015).

Previous studies have shown that employee territorial behavior is influenced by a variety of factors such as individual psychological ownership (X. Chen et al., 2023), negative leadership style (Huang & Tang, 2020), organizational rejection (Deng & Xiang, 2022), and interpersonal relationships. Inclusive leadership, as a positive leadership style that is suitable for Chinese practices, has been proven to stimulate employees’ initiative, suggestion behavior, and other positive performance characteristics (X. Zhu et al., 2025), actively promoting organizational development. However, there are relatively few theoretical and empirical studies exploring how inclusive leadership affects creative territory behavior with both self-protection and innovation attributes, and it is difficult to fully reveal the internal mechanism and potential complexity of inclusive leadership affecting creative territory behavior. Specifically, some scholars (Li & Tang, 2025; Rosing et al., 2011; Zheng et al., 2018) have called for a more systematic examination of how environmental cognition, in conjunction with specific leadership behaviors, affects creative territory behavior (Cao et al., 2023). Self-determination theory points out that people have three basic psychological needs—autonomy, competence, and relationship—and that leaders are the key resource providers to meet employees’ needs. Inclusive leaders have direct effects on employees’ cognition and behavior through these three pathways, which are able to influence employees’ creative territory behavior. Therefore, the mechanism between inclusive leadership and creative territory behavior is clarified, which provides a reference for organizations to correctly apply inclusive leadership behavior, guiding employees to form a positive psychology and avoid creative protective behavior.

Self-determination theory is the core theory that is used to analyze the inner mechanism of individual basic psychological need satisfaction and behavioral motivation stimulation. The harmonious work passion under its framework can explain the transmission path of inclusive leadership on creative territory behavior and supplement the mechanistic perspective based on individual psychological processing. According to self-determination theory, harmonious work passion is a highly autonomous work passion (Zigarmi et al., 2011). It is embodied in the organic integration of the individual’s feelings, cognition, and willingness to work, resulting from the autonomous internalization process of extrinsic motivation, as well as reflecting the degree of integration of work identity into the self (Vallerand et al., 2019). Research shows that the individual harmonious work passion is greatly influenced by the organizational environment in which they are located. Specifically, leadership support (Slemp et al., 2021), mentoring and supportive behavior (Dwertmann & Boehm, 2016; Jansen et al., 2014; Nishii, 2013; Shore et al., 2011), values (Hao et al., 2018; Egan et al., 2019), an inclusive organizational climate (Shore et al., 2011), inclusive behavior among employees (D. Wang et al., 2022), etc., have been proven to be effective in stimulating harmonious work passion among employees. More importantly, harmonious work passions have been widely found to significantly drive multiple positive outcomes for employees. This is reflected not only at the cognitive level (e.g., identification with the organization) and emotional level (e.g., positive emotions and happiness), but also directly in a range of proactive role behaviors (e.g., creativity, proactive behavior, suggestion behavior, etc.) (G. Y. Wang et al., 2016; Vallerand et al., 2007; L. Chen et al., 2021; Ma & Ma, 2021; Vallerand et al., 2003; Lavigne et al., 2012; Yukhymenko-Lescroart & Sharma, 2019). In addition, harmonious work passion also helps to reduce individual negative behaviors, such as self-interested behaviors. Its core mechanism is that harmonious work passion meets the three basic psychological needs of employees, such as autonomy, competence, and belonging, strengthening their organizational belonging and behavior investment level. Therefore, this paper introduces harmonious work passion as a mediator variable to explore the relationship between inclusive leadership and employees’ creative territory behavior.

Based on the “environment–cognition–behavior” model, individual survival and development must be affected by the environment (Locke & Bandura, 1987). In particular, work autonomy, as a key environmental factor, refers to the degree of autonomy and freedom in the selection of work arrangements, contents, methods, and tools (Baer & Frese, 2003). This free environment helps to stimulate employees to optimize and perfect their creativity (Gallego & Kurer, 2022). At the same time, status-competitive motivation, as an individual trait difference variable, reflects the inherent drive of individuals to pursue organizational status (Z. Q. Liu et al., 2013). Whether it is to maintain dignity and face, or to pursue the power, reward, and other resource benefits brought about by status, employees with high status competition motivation usually show more explicit goal orientation and competitive tendencies (Z. Q. Liu et al., 2013). Therefore, when exploring the influencing mechanism of inclusive leadership situations on employees’ creative territory behavior, the interaction of environment and individual traits must be considered. In other words, work autonomy plays a positive role, as an important environmental supporting factor, in the path of inclusive leadership influencing employees’ creative territory behavior, while employees’ status-competitive motivation traits further moderate the effects of environmental factors and leadership style on employee cognition (harmonious work passion) and related behaviors (creative territory behavior).

To sum up, this paper combines self-determination theory, constructs an “environment–cognition–behavior” analysis framework, introduces harmonious work passion factors, and discusses the internal mechanism between inclusive leadership and employee creative territory behavior. At the same time, it investigates the moderating effect of job characteristics (work autonomy) and individual traits (status competition motivation) and their interaction on inclusive leadership and creative territory behavior, which will help optimize the leadership training system, stimulate employees’ innovation enthusiasm, and reduce organizational development difficulties that are caused by territorial consciousness. Additionally, it will provide a scientific basis for differentiated human resource policy formulation, achieving a dynamic balance between employee self-protection and organizational innovation.

## 2. Theoretical Basis and Research Assumptions

### 2.1. Inclusive Leadership and Creative Territory Behavior

Inclusive leadership is a form of relational leadership that invites employees to share ideas and opinions, as well as to listen and pay attention to followers and form unique influences. In the digital age, leaders of this style should be open to the complex internal and external environments, respecting and valuing the differentiated needs of employees (Kong et al., 2020), as well as being willing to listen to and respond to employees and recognize and value their contributions to the organization (Choi et al., 2017). Territorial behavior refers to the behavior expression made by individuals based on the psychological ownership perception of physical or social objects (J. L. Zhang et al., 2017). Based on this, creative territory behavior refers to a series of behaviors whereby individuals construct creative territory scope, declare creative ownership, maintain creative dominance, and recast creative territory control in order to prevent creative territory from being destroyed and invaded (Brown & Robinson, 2005); this behavior includes territorial markers and territorial defense behaviors. Territorial marking refers to the construction and communication of the exclusive ownership of a particular object by an individual or group to other members of the organization; its core lies in communicating territorial boundaries to others. Territorial defense refers to the maintenance and reconstruction of exclusive ownership of an object by an individual or group (Brown & Robinson, 2005). Some studies have shown that organizational context is also an important factor to promote employees to generate creative territory behavior, such as a low trust environment, withholding leadership, paradoxical leadership, and leadership bottom line mentality (Huang & Tang, 2020; Tan & Yuan, 2024). Therefore, as a special organizational context that is directly related to employees, inclusive leadership may affect creative territory behavior.

Self-determination theory points out that people have three basic psychological needs—autonomy, competence, and relationship. Creative territory behavior originates from the conflict between employees’ ownership of ideas and their organizational openness needs, while leaders are key resource providers to meet needs. Inclusive leaders have an effect on employees’ cognition and behavior through a triple-pronged pathway, reconstructing employees’ cognition of creative ownership, as well as influencing their creative territory behavior and making them tend to share rather than monopolize ideas (Avey et al., 2009). Firstly, in the face of complex internal and external environments in the digital age, inclusive leaders can respect and value the differentiated needs of employees with an open attitude (Kong et al., 2020), identify individual differences in employees (Bradley, 2020), and provide personalized resource support to enhance their ability identification so that employees are more willing to prove their own value through cooperation rather than exclusive creativity. Inclusive leaders enhance employees’ sense of “ownership responsibility” by valuing and supporting employee behaviors (Su et al., 2023); as such, employees are more likely to think in terms of organizational development. Secondly, the interaction between leaders and employees has the characteristics of openness, effectiveness, and accessibility (J. Zhong et al., 2018) in order to satisfy employees’ autonomous needs and encourage them to apply new perspectives and new approaches to organizational problems (Mir et al., 2021), reduce the sense of control over creative sharing, and provide rich media channels for communication and exchange among organization members. The friendship between employees is gradually strengthened. When the friendship between organizations is strengthened, employees are more willing to share their own ideas and knowledge in the enterprise (Guo et al., 2024; Jia et al., 2022). At the same time, employees obtain support and help from leaders; inclusive leaders pay attention to employees’ needs and interests, and as important situational factors affecting employees’ knowledge decision-making, they significantly negatively predict knowledge hiding behavior (X. Zhong et al., 2019, 2020; Wen et al., 2022). Knowledge hiding and creative territory behavior are both derived from protective motivation for one’s own resources. As employees’ own resources are constantly replenished and enriched, employee initiative is enhanced (Xu et al., 2021; Kong et al., 2020), reducing exclusivity or territorial behavior resulting from differentiating creative resources (B. Cheng et al., 2023). Finally, inclusive leaders are better able to accept employees’ different opinions and tolerate their work mistakes (Qi et al., 2019), as well as providing more support for employees with a tolerant attitude, fostering diverse teams and shaping an inclusive culture of the organization (Nishii, 2013), positively affecting team members’ sense of belonging and being valued (Hundschell et al., 2025). In such an environment, communication barriers between teams are low, which, in turn, enhances team cohesion and cooperation (Nishii & Mayer, 2009), which further translates into employee willingness to participate in organizational development. Employees believe that sharing ideas is a matter of promoting team progress and personal development, as well as contributing to the organization through proactive behaviors such as creative sharing (George & Zhou, 2002). Inclusive leadership thus influences employees and the organizational climate to share ideas and to reduce creative territory. Based on the above analysis, this paper proposes the following assumptions:

**H1.**  *Inclusive leadership negatively affects creative territory behavior.*

### 2.2. Mediating Effect of Harmonious Work Passion

There is duality in work passion, among which harmonious work passion refers to employees’ heartfelt love for work, willingness to devote themselves to work, and their autonomy and flexibility in dealing with work (Vallerand et al., 2003). According to self-determination theory, harmonious work passion is a form of highly autonomous work passion (Zigarmi et al., 2011). It is the integration of individual emotion, cognition, and intention, which occurs in the process of internalizing extrinsic motivation (Vallerand et al., 2019). Previous studies have shown that employee characteristics (such as autonomy tendency, self-regulation mode, and control point), organizational environment (such as leadership style, autonomy support, and cooperative psychological atmosphere), and job characteristics (such as responsibility ambiguity, autonomy, performance feedback, and task diversity) are the main influencing factors of job passion (Philippe et al., 2009; Zigarmi et al., 2018; Wu et al., 2021). Based on this, this paper concludes that inclusive leadership, as an important external work resource, is conducive to employees’ harmonious work passion.

Self-determination theory holds that inclusive leadership, as a relational leadership style, provides employees with abundant resources, respects differences, recognizes employees’ individual values, and encourages them to give full play to their strengths (Zhao et al., 2020). Inclusive leadership creates a positive atmosphere that also meets employee relationship needs. Firstly, inclusive leaders are willing to listen and respond to employees (Choi et al., 2017; Ahmed et al., 2020). Employees realize that they are an integral part of the team, which, in turn, enhances their sense of belonging at work (Randel et al., 2018). This sense of belonging will lead to higher levels of harmonious work passion among employees. Secondly, employees receive support and respect from leaders (Hao et al., 2018; Egan et al., 2019) in order to meet the individualized resource needs of employees in dealing with difficulties and challenges at work (Randel et al., 2018; B. C. Wang et al., 2025). This kind of support enhances the employee’s own sense of security and ensures they have more room for growth under this kind of support; therefore, employees will be more likely to have a harmonious work passion. Finally, employees’ harmonious work passion is influenced by the external environment and produces positive cognition and emotion (Mueller et al., 2017). The effect of organizational support (Y. J. Jiang et al., 2017) means that employees’ psychological needs are met and everyone in the organization is encouraged to remain authentic to themselves (Dwertmann & Boehm, 2016; Jansen et al., 2014; Nishii, 2013; Shore et al., 2011) and promote positive team relationships (Zheng et al., 2018). As a result, employees obtain satisfaction and pleasure from work itself and promote the development of harmonious passion (D. Wang et al., 2022). Based on the above analysis, this paper proposes the following assumptions:

**H2.**  *Inclusive leadership positively affects employees’ harmonious work passion.*

Harmonious work passion can largely satisfy employees’ psychological needs (Vallerand et al., 2003). According to self-determination theory, employees with high levels work passion of the harmonious type reconstruct their cognition of creative ownership in order to meet their own needs for competence, autonomy, and relevance in order that they regard work as a way of self-realization rather than a competitive tool, thus weakening the defensive motivation to protect creativity and strengthening collaborative sharing, subsequently reducing creative territory behavior. Specifically, employees with high levels of harmonious passion consciously invest time and effort in cognitive and evaluative processes (Zigarmi et al., 2018), creating a sense of self-determination and self-acceptance. Research has shown that harmonious work passions are often associated with positive work outcomes in work settings, such as job satisfaction, flow experience, and creativity (Slemp et al., 2021). Therefore, harmonious work passion can stimulate positive emotions and immersive experiences in employees (Lavigne et al., 2012; Yukhymenko-Lescroart & Sharma, 2019). Employees with a harmonious work passion will experience individual value and meaning in their work; such employees regard work as an extension of themselves (Perrewé et al., 2014). They show strong positive emotions and internal motivation for work, as well as paying more attention to the overall value of ideas to the organization rather than to individual monopoly gains (Y. Yang et al., 2025). This reduces self-protection at work. In addition, employees with a harmonious work passion continuously improve their own qualities, such as actively participating in training and learning, increasing their knowledge reserves, etc., (Ho & Astakhova, 2018). Their heartfelt love for work motivates them to actively participate in work and pay attention to new ideas and measures that are conducive to improving organizational efficiency and promoting organizational development. In this state, employees are more willing to break down knowledge barriers and share their ideas. Finally, employees with a harmonious work passion will increase individual positive behaviors to bring about additional benefits to the organization (J. C. Zhang & Ling, 2016). They will put more energy into work and will pay more attention to achieving work goals through active cooperation; work value will be tied to organizational goals rather than gaining a sense of achievement by defending creative territory. Therefore, they will reduce the levels of creative territory behavior. Based on the above analysis, this paper proposes the following assumptions:

**H3.**  *Harmonious work passion negatively affects creative territory behavior.*

Inclusive leadership positively affects harmonious work passion by satisfying employees’ psychological needs, while harmonious work passion negatively affects creative territory behavior. Research has shown that leadership, as a key component of the environment in which employees are located, affects their harmonious passions and activates corresponding behaviors (G. Yang & Xiao, 2025). Role theory also suggests that harmonious work passions encourage employees to focus more on the overall interests of the team and reduce the overprotection of individual creative territories (Biddle, 1979). According to the process of “job characteristics–individual emotion–behavior”, inclusive leadership, as an important source of job characteristics, promotes the development of harmonious work passion by meeting employees’ needs for autonomy, competence, and belonging (D. Wang et al., 2022). Employees with a harmonious work passion will experience individual value and meaning in their work, show strong positive emotions and internal drive for work, consider fewer material benefits and results brought about by work, and be more willing to invest time and energy in work. At this time, employees with harmonious work passion will continuously improve their own qualities in order to complete their work with higher quality (Ho & Astakhova, 2018), accepting and integrating positive organizational behavior, initiative behavior, change initiation behavior, and suggestion behavior into the individual identity system (L. Chen et al., 2021; Ma & Ma, 2021), thereby reducing unsanctioned territorial behavior in the organization. Based on the above analysis, this paper proposes the following assumptions:

**H4.**  *Harmonious work passion mediates the negative effects of inclusive leadership and creative territory behavior.*

### 2.3. The Moderating Role of Work Autonomy

In the organizational context, work autonomy refers to the degree of autonomy and freedom an employee has to independently arrange work content, tools, and methods, including the discretion to independently arrange work content and the substantive freedom to independently determine work procedures (Baer & Frese, 2003). Therefore, work autonomy, as an important job characteristic in organizations, has a certain influence on employees’ emotions and behaviors. At the individual level, giving employees greater autonomy in their work fosters a greater sense of responsibility, growth, and self-fulfillment.

Based on the self-determination theory, greater work autonomy leads to a greater sense of control over work processes and plans, as well as a greater sense of self-achievement. As an environmental variable, harmonious work passion is enhanced by strengthening inclusive leadership to meet autonomous needs. Inclusive leadership can provide more room for employees to exercise their own choices in situations with high work autonomy (Kong et al., 2020; B. C. Wang et al., 2025; Arnold & Bakker, 2016). Employees will feel more strongly that their work is internally motivated because their autonomous needs are being adequately met (B. C. Wang et al., 2025) and initiative is stimulated (Khattak et al., 2022). As such, they are able to work according to their own wishes, so as to be more proactive in their work. This internal motivation stimulates employees to have more enthusiasm for their work, which, in turn, enhances harmonious work passion. The “job demands–resources” model also points out that job resources help employees cope with job demands, reduce job stress, and promote personal growth and development. Work autonomy is an important work resource (B. C. Wang et al., 2025) and inclusive leadership provides employees with an abundance of personalized resources. Inclusive leadership creates a positive climate and provides support when work autonomy is high (Shore et al., 2011); these characteristics complement autonomy. Employees can take advantage of higher work autonomy to better display their abilities in an inclusive leadership environment, transform the resources provided by leaders into their own growth and development, and further enhance their enthusiasm for work.

On the contrary, when the autonomy of work is low, even if the leader is inclusive, employees may not be able to use the right of independent choice, resulting in unsatisfied autonomy needs and difficulty in generating strong internal motivation; in this situation, employees have relatively limited resources available to them (B. C. Wang et al., 2025). Unable to give full play to their own abilities, it is difficult to obtain a sufficient sense of achievement from their work, and the promotion of inclusive leadership to harmonious work passion will be weakened. Based on the above analysis, this paper proposes the following assumptions:

**H5a.**  *Work autonomy positively moderates the relationship between inclusive leadership and harmonious work passion. The higher the work autonomy, the more significant the positive correlation between inclusive leadership and harmonious work passion, as well as the weaker the correlation between inclusive leadership and harmonious work passion.*

**H5b.**  *Work autonomy positively moderates the indirect effect of inclusive leadership on creative territory behavior through harmonious work passion. The higher the work autonomy, the more significant the indirect influence of harmonious work passion, as well as the weaker the inverse.*

### 2.4. Moderating Effect of Status Competition Motivation on Work Autonomy

Status, as a symbol, can bring satisfaction to people, such as high-quality face, dignity, reputation, and prestige (Z. Q. Liu et al., 2013). Status competition motivation reflects employees’ desire to gain influence in their organization because status can symbolize face, dignity, etc., (Z. Q. Liu et al., 2013). It has the function of capability signaling, location resourcing, and has a low-cost exchange (Tan & Yuan, 2024). In status competition, if employees value the resources behind status more, dominant status competition motivation will be formed (Y. Chen et al., 2012; Bendersky & Hays, 2012); this motivation is position-seeking-oriented, expecting to dominate resources and others, seeking more control, which is a typical possessive pursuit. If employees pay more attention to the symbolic meaning of status itself, it will form a position-based competitive motivation that is based on prestige (Y. Chen et al., 2012; Bendersky & Hays, 2012), which is a typical symbolic pursuit that is oriented towards status maintenance, expecting recognition and acceptance from others, and striving to maintain a perfect image in the eyes of others.

According to self-determination theory, status-based competitive motivation can activate employees’ competence needs. When employees with status-competitive motivation perceived higher work autonomy, their autonomous needs were better satisfied, they could choose work styles and goals more autonomously, and they experienced stronger intrinsic motivation. Social cognitive theory also holds that there is a dynamic relationship between individuals, their behavior, and the environment in which they are located; individual behavior decision-making depends on the joint action of environmental factors and individual factors, to some extent (Locke & Bandura, 1987). Specifically, more sustainable intrinsic motivation occurs when individual behavior is aligned with intrinsic will. Employees with a high level of dominant competitive motivation take control of others and resources as their core goal, and their behavior essence is to control their goals through independent decision-making and influencing the environment. Therefore, they have a strong demand for work autonomy. The higher the autonomy, the higher the satisfaction of their autonomy needs, and the stronger the consistency between behavior and intrinsic motivation. On the other hand, employees with high levels of competitive motivation for authoritarian status satisfy their competency needs by demonstrating authority and competitive status, essentially by demonstrating their ability and influence. Such behavior signals to others that they are in control and trustworthy, gaining recognition for their work and increasing self-efficacy. The higher the work autonomy, the greater the sense of control employees feel over their work, reinforcing their internal motivation. Inclusive leaders give employees a sense of belonging and psychological support (Shore et al., 2011), letting them plan their work according to their own ideas and abilities. For status-motivated employees, this not only satisfies their need for autonomy, but also gives them the opportunity to demonstrate their mastery at work in order to gain higher status recognition, which, in turn, enhances their passion for work. Harmonious work passion makes employees more willing to cooperate with others and share ideas, thus reducing defensive behaviors to protect creative territory. Inclusive leadership has a significant negative impact on creative territory behavior through harmonious work passion.

Conversely, when such employees have low levels of perceived autonomy, even if their leaders are inclusive, they will be unable to generate strong intrinsic motivation because their autonomy needs are not fully met and resources are limited. The positive influence of inclusive leadership on harmonious work passion will be weakened if employees cannot bring their abilities into full play and obtain enough sense of achievement from work to meet their needs. They may be more inclined to protect their limited resources and opportunities by maintaining their creative territory to satisfy their status-competitive motives. At this time, the negative impact of inclusive leadership on creative territory behavior through harmonious work passion will be weakened. Based on the above analysis, this paper proposes the following assumptions:

**H6.**  *Inclusive leadership, work autonomy, and status competition motivation have a three-dimensional interaction relationship with harmonious work passion. The higher the perceived work autonomy of employees with status-competitive motivation, the more significant the positive effect of inclusive leadership on harmonious work passion, and the more significant the indirect effect of inclusive leadership on creative territory behavior through harmonious work passion, and the weaker the reverse.*

**Figure 1:**
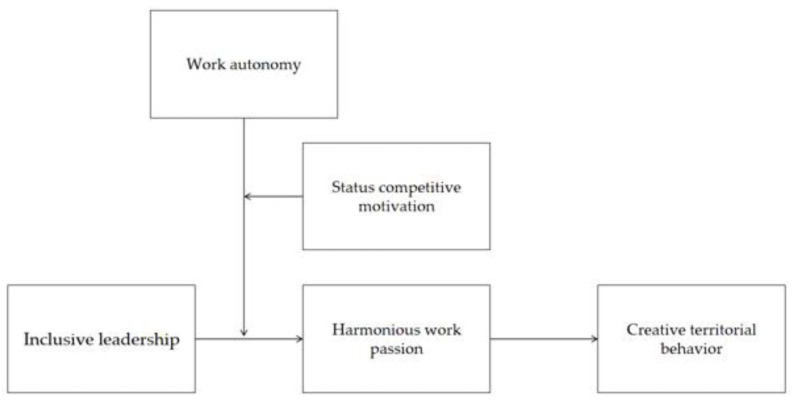
Research model.

The research model of this paper is shown in Figure 1.

## 3. Study 1: Scenario Experiment

### 3.1. Study Methods

#### 3.1.1. Study Samples

G*Power 3.1 software was used to calculate the sample size required for the experiment. For the two-way ANOVA applicable to this experiment, the medium effect size f = 0.25, the significance level α = 0.05, the number of groups was 4, and at least 179 subjects were required to achieve an 80% statistical test power (Faul et al., 2009). We recruited 240 MBA students through social connections and project group resources; after excluding samples that failed the attention test, the final sample size included 227 participants. (Please select A. Very dissatisfied; B. Fair; or C. Very dissatisfied and strictly screen the seriousness of the participants‘ answers.) Among them, 119 were male (accounting for 52.42%) and 108 were female (accounting for 47.58%). The age distribution was as follows: 58 (accounting for 25.55%) were aged 26–30; 120 (accounting for 52.86%) were aged 31–35; 30 (accounting for 13.22%) were aged 36–40; and 19 (accounting for 8.37%) were aged over 41.

#### 3.1.2. Experimental Design and Procedure

The experiment adopted a 2 (inclusive leadership: yes vs. no) × 2 (work autonomy: yes vs. no) two-factor between-group design, including four experimental situations. All participants were randomly assigned to one of four groups and asked to imagine themselves as employees in a work team. At the beginning of the experiment, participants were asked to imagine their supervisor (Li, in the experimental scenario) as much as possible and to read the situational materials related to inclusive leadership and work autonomy. After reading, they were asked to recall and describe the contents they had read for no less than 5 min. Participants then completed measures of inclusive leadership, work autonomy, harmonious work passion, creative territory behavior, and demographic variables.

Inclusive leadership manipulation (scenario materials adapted from the content developed by Rego et al. (2019).

Imagine that you are Xiao Wang, the protagonist, currently engaged in technology research and development in an emerging technology enterprise, mainly involving the integrated operation business of intelligent terminals such as digital products from R&D, production, and marketing to operation. The company has a good development momentum and is a stable business. You have been working in this company for 5 years. At this stage, you are working as a customer service manager, covering customer complaints, customer orders, and customer information maintenance and processing.

High Inclusive Leadership: Manager Li is willing to listen to new ideas or solutions in his daily work, actively pay attention to new opportunities that can improve work processes, and is willing to discuss organizational vision and ways to achieve it with you. If you have problems at work, you can always ask him for advice, and he has always been in the team and can be found at any time. When you want to consult Manager Li about professional problems, you can also communicate with him in a timely manner. In general, Manager Li is willing to listen patiently to your ideas and suggestions, encourages you to discuss problems with him when you find problems in your daily work, and always finds ways to discuss new solutions and new problems together.

Low Inclusive Leadership: Manager Li is unwilling to listen to new ideas or solutions in his daily work and does not pay attention to new opportunities that can improve work processes. He is usually unwilling to discuss the organization vision and ways to achieve it with you. If you have a problem at work, you cannot always ask him for advice, and he is not always on the team. When you want to consult Manager Li about professional problems, you cannot find him at any time. In general, Manager Li is not willing to listen to your ideas and suggestions patiently, and you are not encouraged to discuss problems with him when you find them in your daily work. You cannot find him to discuss new solutions and new problems together.

Autonomy at work (scenario material adapted from Breaugh’s 9-item Work Autonomy Scale (Breaugh, 1999)).

High Work Autonomy: You can choose your own customer complaint solution (e.g., direct replacement, compensation, or remotely guided repair) and adjust your communication style to customer personality (e.g., for a technical customer, you can detail the principles or for an older customer, you can simplify the steps). You can freely adjust the order of work order processing, such as placing VIP customers or batch problems to the top, or even suspend non-urgent tasks to focus on crisis events. You have flexibility in setting service targets, e.g., for repeat complaints, and you can initiate additional compensation without prior approval.

Low work autonomy: Although the company has a good momentum of development, in order to improve service standardization, recently, the management has tightened the work authority of the customer service department and has implemented strict process controls. You must strictly follow the Customer Complaint Handling Manual formulated by the company. Each step (such as pacification, problem classification, and handover process) must be executed according to a fixed template and cannot be flexibly adjusted according to the actual situation of the customer. Customer complaint work orders are automatically assigned and forced by the system, and you must process them in order, even if you encounter urgent problems (such as batch product failures). Performance appraisal is only based on the “work order closing rate” and “average processing time”. Even if the customer puts forward reasonable requirements (such as extending the after-sales period), you have no right to additional assistance and need to ask for instructions step by step.

#### 3.1.3. Variable Measurement

A Likert 5-point scoring method was used for all items of the scale, from 1 to 5 representing “strongly disagree” to “strongly agree”, respectively.

(1) Inclusive leadership. This study uses a scale developed by Carmeli et al. (Carmeli et al., 2010) that contains nine items. Typical items are “My supervisor is willing to discuss with me sudden problems in work”, “My supervisor is willing to provide consultation on work-related problems”, etc. Its Cronbach’s alpha coefficient is 0.948.
(2) Harmonious work passion. This system is based on Vallerand and Houlfort’s self-report scale and Chen et al.’s other-rating scale (Vallerand et al., 2003). Its Cronbach’s alpha coefficient is 0.875.
(3) Creative territory behavior. A one-dimensional 4-item scale developed by Avey et al. (Avey et al., 2009) was used that contains typical items such as “feeling the need to protect one’s innovative ideas from others in the organization”. Its Cronbach alpha coefficient is 0.894.
(4) Autonomy of work. This study uses the 9-item scale from the Breaugh Study (Breaugh, 1999). Typical items are “I can plan and arrange my work according to my own wishes” and “My work allows me to make my own decisions”. Its Cronbach’s alpha coefficient is 0.943.

### 3.2. Research Results

#### 3.2.1. Control Check

Independent sample t-test results showed that the high levels of inclusive leadership group (M = 4.01, SD = 0.93) was significantly higher than the low levels of inclusive leadership group (M = 2.38, SD = 0.88) (t(225) = −13.594; *p* < 0.001), indicating that the experiment was successful in manipulating inclusive leadership. For the manipulation of work autonomy, the independent sample t-test results showed that the high levels of work autonomy group (M = 4.17, SD = 0.60) was significantly higher than the low levels of work autonomy group (M = 2.60, SD = 1.03) (t(225) = −14.037; *p* < 0.001), indicating that the manipulation of work autonomy was successful.

#### 3.2.2. Confirmatory Factor Analysis

Confirmatory factor analysis (CFA) was performed with Mplus 8.0 on core variables to test the fitness of the model and the discriminative validity of the variables. The results are shown in Table 1. The fitting degree of the four-factor model is good (χ^2^ = 406.290; df = 371; χ^2^/df = 1.095; CFI = 0.992; TLI = 0.991; RMSEA = 0.021; SRMR = 0.040) and it is significantly better than other alternative models, indicating that the model has good structural validity and discrimination validity for each variable.

#### 3.2.3. Hypothesis Testing

The mean, standard deviation, and correlation results of the variables are shown in Table 2. Participants in the high levels of inclusive leadership group reported significantly lower levels of creative territory behavior (M = 3.04, SD = 1.21) than participants in the low levels of inclusive leadership group (M = 3.82, SD = 0.97) (t(225) = 5.39; *p* < 0.001; Cohen’s d = 1.096). Therefore, inclusive leadership has a significant negative impact on employees’ creative territory behavior; assumption H1 gains support.

Participants in the high levels of inclusive leadership group reported a significantly higher level of harmonious work passion (M = 4.00, SD = 0.90) than participants in the low levels of inclusive leadership group (M = 3.26, SD = 0.99) (t(225) = −5.818; *p* < 0.001; Cohen’s d = −0.773). Therefore, inclusive leadership has a significant positive impact on harmonious work passion; assumption H2 obtains support.

The linear regression model from SPSS 29.0 was used to test Hypothesis 3, Table 3. According to the regression results of model 2, harmonious work passion had a significant negative impact on employees’ creative territory behavior (B = −0.511 ***, *p* < 0.001) and H3 was supported.

Inclusive leadership (low group vs. high group) was taken as an independent variable, employee harmonious work passion was taken as a mediating variable, and creative territory behavior was taken as a dependent variable. Bootstrap times were set as 5000, and a bias-adjusted 95% confidence interval was used to test the mediating effect. The results show that the mediating effect of harmonious work passion is −0.315, while the 95% bootstrap confidence interval is [−0.204, −0.078]. This does not contain 0, indicating that the mediating effect is significant, and H4 is supported.

**Figure 2:**
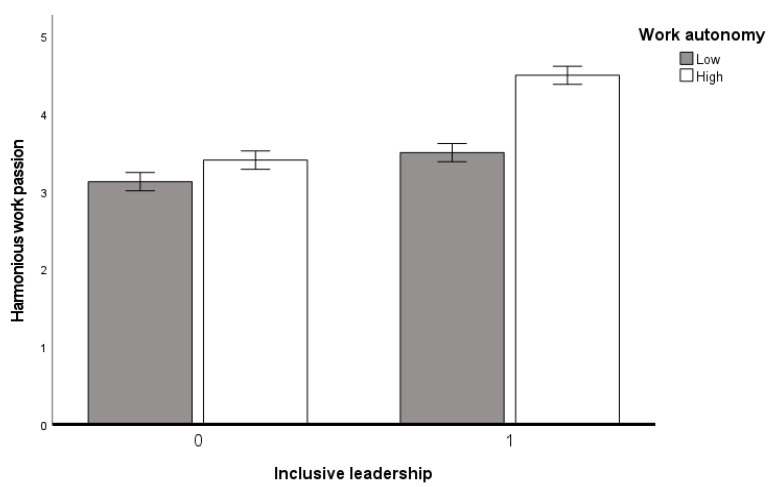
Interaction between inclusive leadership and work autonomy.

A two-way ANOVA analysis was conducted with harmonious work passion as the dependent variable, Figure 2. It was found that there was a significant interaction between inclusive leadership and work autonomy (F(1,223) = 9.355; *p* < 0.01; ηp^2^ = 0.040; 95%CI = [0.255,1.177]). As shown in Figure 2, in the low levels of inclusive leadership scenario, employees with high levels of work autonomy have higher levels of harmonious work passion (M = 3.398, SD = 0.988) than in the low levels of inclusive leadership scenario (M = 3.120, SD = 0.0.991) (t(225) = −6.598; *p* < 0.001; Cohen’s d = 0.163). In the high levels of inclusive leadership scenario, employees’ harmonious work passion under the condition of high levels of work autonomy (M = 4.488, SD = 0.488) was different from that under the condition of low levels of work autonomy (M = 3.308, SD = 0.946) (t(225) = −6.04; *p* < 0.001). Therefore, the hypothesis H5a is verified.

Inclusive leadership (low group vs. high group) was taken as independent variable, employee harmonious work passion as mediating variable, creative territory behavior as dependent variable, work autonomy (low group vs. high group) as moderating variable. Bootstrap times were set at 5000, and 95% confidence interval with bias correction was used to test the moderated mediating effect, Table 4. The results showed that the mediating effect of harmonious work passion was −0.467, 95% CI = [−0.685,−0.274] did not contain 0 under the condition of low work autonomy, and the mediating effect of harmonious work passion was −0.162, 95% CI = [−0.326,−0.009] did not contain 0 under the condition of high work autonomy, indicating that the mediating effect was significant. In conclusion, H5b is assumed to be supported.

Study 1 examined the causal relationship between the interaction of inclusive leadership, work autonomy, harmonious work passion, and creative territory behavior using a situational experiment, which enhanced the internal validity of the study. However, considering that there may be some differences between the simulated experimental situation and the real work situation, the external validity of the study’s conclusions needs to be expanded. Study 1 did not verify the three interactive effects of inclusive leadership, work autonomy, and status competition motivation. Therefore, in Study 2, the overall model was validated using a multi-point questionnaire survey.

## 4. Study 2: Questionnaire Survey

### 4.1. Data Collection and Research Samples

Because this paper discusses the influence of inclusive leadership on employees’ creative territory behavior, the sample data within this paper are selected from IT, finance, manufacturing, and energy industries in Shandong, Xinjiang, and Henan. These industries were chosen for data collection because their external environments change relatively quickly and require companies to adapt to dynamic competitive environments through increased creativity. Questionnaire distribution was adopted in two ways—offline and online. Offline, paper questionnaires were distributed to MBA students by contacting enterprises and institutions where friends are located. Online, questionnaires were sent through QQ, WeChat, Weibo, and other social platforms.

In T1 (January 2025), employees were required to record basic personal information (including age, gender, education, working years, etc.), inclusive leadership, work autonomy, and status competition motivation. In T2 (March 2025), employees were required to record harmonious work passion and creative territory behavior. In order to facilitate the matching of the two rounds of questionnaires, the subjects were asked to fill in the last four digits of their mobile phone number and were reminded that the two rounds should be consistent; otherwise, it would be considered invalid. After each questionnaire, employees had the opportunity to receive red envelopes ranging from CNY 5 to 20. In order to reduce the turnover rate of the follow-up sample survey, after the questionnaire survey occurred, the follow-up survey QR code is attached. If employees want to participate in the follow-up research, they can voluntarily enter the WeChat group. In T1, 860 employees were surveyed, and 781 valid questionnaires (effective rate: 90.81%) were obtained after eliminating invalid questionnaires. In T2, 689 questionnaires were collected, and 570 valid questionnaires (effective rate: 82.73%) were obtained after eliminating invalid questionnaires. After the two rounds of questionnaires, those with regular answers or unmatched questionnaires were eliminated. As such, 461 valid questionnaires were retained. The sample details are shown in Table 5.

### 4.2. Measuring Tool

A Likert 5-point scoring method was used for all items of the scale, ranging from 1 to 5, which represent “strongly disagree” to “strongly agree”, respectively.

(1) Inclusive leadership. This study uses a scale developed by Carmeli et al. (Carmeli et al., 2010) that contains nine items. Typical items are “My supervisor is willing to discuss with me sudden problems in work”, “My supervisor is willing to provide consultation on work-related problems”, etc. Its Cronbach’s alpha coefficient is 0.948.
(2) Harmonious work passion. This evaluation is based on Vallerand and Houlfort’s self-report scale and Chen et al.’s other-rating scale (Vallerand et al., 2003). Its Cronbach’s alpha coefficient is 0.875.
(3) Creative territory behavior. A one-dimensional 4-item scale developed by Avey et al. (Avey et al., 2009) was used, containing typical items such as “feeling the need to protect one’s innovative ideas from others in the organization”. Its Cronbach alpha coefficient is 0.894.
(4) Autonomy of work. This study uses the 9-item scale from the Breaugh Study (Breaugh, 1999). Typical items are “I can plan and arrange my work according to my own wishes” and “My work allows me to make my own decisions”. Its Cronbach’s alpha coefficient is 0.943.
(5) Motivation for status competition. Cheng et al.’s (J. T. Cheng et al., 2013) scale was used, with representative items such as “I often strive to achieve my goals regardless of what the rest of the team thinks”. Its Cronbach’s alpha coefficient is 0.947.
(6) Control variables. Following the recommendations of previous studies (B. C. Wang et al., 2025; J. Jiang et al., 2022; Zheng et al., 2018) and in order to rule out other factors affecting employee creative territory behavior, we controlled for individual variables, including employee gender, age, education, position, tenure, and industry.

### 4.3. Data Analysis and Hypothesis Testing

#### 4.3.1. Common Method Bias

In this paper, the questionnaire survey uses a form of employee self-evaluation. Although it uses regional zoning, anonymity, and other pre-control methods, it is still subject to common method bias. Therefore, common method bias testing is required prior to data analysis. According to Harman’s one-way factor analysis, exploratory factor analysis was carried out on all items using SPSS29.0 software, and five factors were separated out without rotation. Among them, the first factor explained 31.618% of the total variance, which was less than the critical value of 40%. Therefore, there was no significant common method bias effect among the relevant variables in this study, which was verified by subsequent analysis.

#### 4.3.2. Confirmatory Factor Analysis

Confirmatory factor analysis was used to test the validity of the scale. The KMO coefficient measured was 0.963, which was greater than 0.8 (*p* = 0.000); Bartlett’s spherical test was significant. The results of the confirmatory factor analysis are shown in Table 6. It can be seen that the fitting indexes of the basic model are χ^2^/df = 1.357, which are less than 3; RMSEA = 0.028 and SRMR = 0.032, both of which are less than 0.05; the TLI and CFI values are 0.974 and 0.975, respectively, both of which are greater than 0.9 and within the required range, indicating that the model fits well and is obviously better than the four-factor model, three-factor model, two-factor model, and one-factor model. Therefore, it has sufficient discrimination validity.

#### 4.3.3. Descriptive Statistics and Correlation Analysis

The mean, standard deviation, and correlation coefficient results for each study variable are shown in Table 7. The results showed that inclusive leadership was negatively correlated with creative territory behavior (r = −0.494, *p* < 0.05); inclusive leadership was positively correlated with harmonious work passion (r = 0.382, *p* < 0.01); and harmonious work passion was negatively correlated with creative territory behavior (r = −0.451, *p* < 0.05).

#### 4.3.4. Hypothesis Testing

(1) Mediating effect test

The linear regression model of SPSS 29.0 was used to test both the main effects and the intermediate effects. The results are shown in Table 8. According to Model 6, inclusive leadership is negatively correlated with creative territory behavior (β = −0.478, *p* < 0.001) and H1 is verified. According to Model 9, inclusive leadership is significantly positively correlated with harmonious work passion (β = 0.381, *p* < 0.001) and hypothesis H2 is verified. According to model 7, harmonious work passion is significantly positively correlated with creative territory behavior (β = −0.308, *p* < 0.001) and H3 is verified. The regression coefficient between inclusive leadership and creative territory behavior is −0.361 (*p* < 0.001); H4 is supported.

In addition, the mediating effect of harmonious work passion between inclusive leadership and creative territory behavior was explored through the process mediating effect test, and a 95% unbiased-correction confidence interval was constructed. The test results after repeated sampling of 5000 times are shown in Table 9. In terms of the direct effects, the effect of inclusive leadership on creative territory behavior is −4.026, with a 95% confidence interval of [−4.939, −3.112], excluding zero. Assumption H1 is supported; the relative effect is 75.45%. For the indirect effects, the effect value is −1.311, with a 95% confidence interval of [−1.772, −0.0909], which does not contain zero, indicating that the mediating effect is significant. Assumption H4 holds; the relative effect value reaches 24.57%. The above steps verify that harmonious work passion mediates the negative correlation between inclusive leadership and creative territory behavior.

(2) Mediating effect test

A Test of the Moderating Role of work autonomy.

In order to test the significance of the interaction coefficient, the control variable, independent variable, and interaction term were added into the regression equation in turn. Control variables such as gender, age, education, position, tenure, and industry were included in Model 10. Model 12 was constructed by incorporating all the second-order interaction terms in Model. Model 13 was constructed by introducing the interaction terms of inclusive leadership × work autonomy × status competition motivation.

**Figure 3:**
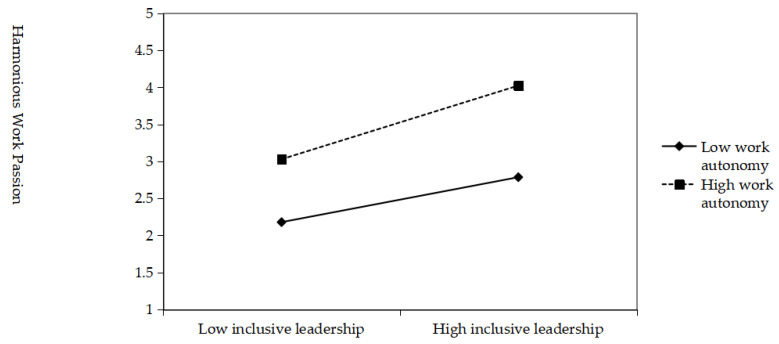
The moderating effect of work autonomy.

In this paper, the hierarchical linear regression model is used to test the moderating effect of task complexity, and the results are shown in Table 10. A moderating effect diagram was drawn to clearly show the moderating effect; the results are shown in Figure 3. From Model 3, it can be seen that the interaction between inclusive leadership and work autonomy has a positive effect on harmonious work passion (β = 0.159, *p* < 0.001), indicating that work autonomy positively regulates the relationship between inclusive leadership and harmonious work passion; assumption H5a holds.

Figure 3 shows that higher levels of work autonomy enhance the positive impact of inclusive leadership on harmonious work passion compared with lower levels of work autonomy; i.e., work autonomy positively modulates the relationship between inclusive leadership and harmonious work passion; assumption H5a is further confirmed. In addition, the bootstrap method was used to test the moderated mediation effect, and the results are shown in Table 11. The results showed that the mediating effect of harmonious work passion was −0.1740 and−0.0317, the difference was−0.1423, and the 95% confidence intervals were [−0.2342, −0.1205], [−0.0775, 0.0127], and [−0.2126, −0.0771], respectively. At the high and low levels of work autonomy, the high level and the difference were significant and did not include 0. However, the indirect effect value increases significantly with the increase in work autonomy, and inclusive leadership has a stronger effect on creative territory behavior through harmonious work passion. The low level includes 0, which indicates that the effect is not significant, i.e., the mediation utility is weaker or even disappears when the work autonomy level is low. H5b is verified.

Interaction among work autonomy, inclusive leadership, and motivation point of status competition.

**Figure 4:**
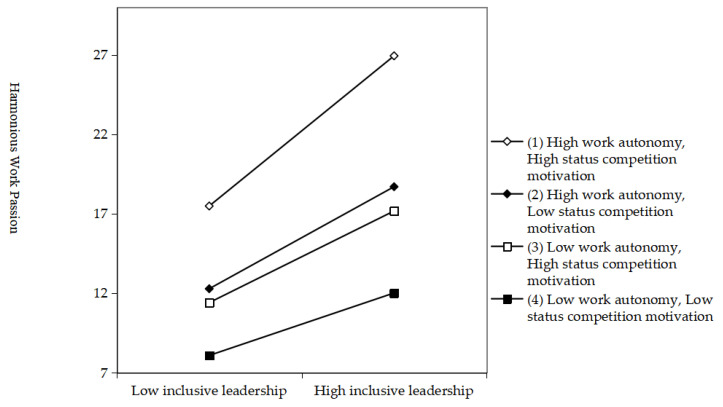
The interactive moderating effect of work autonomy and status competition motivation.

According to Model 13 in Table 10, there is a significant positive correlation between the three interaction items and harmonious work passion (β = 0.166, *p* < 0.001). This indicates that under inclusive leadership, employees with high levels of status-competitive motivation have higher levels of harmonious work passion as their work autonomy increases. To further verify H6, according to Dawson and Richter’s (Dawson & Richter, 2006) research method, the combination of work autonomy and status competition motivation is subdivided into four situations—low work autonomy and low status competition motivation, low work autonomy and high status competition motivation, high work autonomy and low status competition motivation, and high work autonomy and high status competition motivation, Figure 4. It can be seen that, under the condition of high work autonomy and high status competitive motivation, the positive slope of the curve fitting the influence of inclusive leadership on harmonious work passion is the largest, which means that the positive influence of inclusive leadership on harmonious work passion is the most significant under this condition.

Bootstrap testing for moderated mediating effects was continued, as shown in Table 12. The results showed that the 95% confidence intervals were [−0.0625, 0.0314], [−0.0899, 0.0651], and [−0.1158, −0.0721] under the motivation of low work autonomy and low status competition, low work autonomy and high work autonomy, and low status competition, respectively, all of which contained zero, indicating that inclusive leadership had no significant effect on creative territory behavior through harmonious work passion. Under the conditions of high work autonomy and high status competitive motivation, the 95% confidence interval does not contain 0, and the mediating effect of harmonious work passion increases significantly. Therefore, inclusive leadership has the most significant indirect effect on creative territory behavior through harmonious work passion when employees with high levels of status competitive motivation accept high levels of work autonomy. In conclusion, H6 is verified.

## 5. Conclusions and Discussion

### 5.1. Research Conclusions

Based on self-determination theory, this study introduces inclusive leadership, work autonomy, and status competition motivation into the analysis framework of the “environment–cognition–behavior” process in order to explore the internal mechanism and boundary conditions of inclusive leadership affecting employees’ creative territory behavior. The results show that inclusive leadership negatively affects creative territory behavior; harmonious work passion mediates the negative effects of inclusive leadership and creative territory behavior; work autonomy positively moderates the relationship between inclusive leadership and harmonious work passion; and inclusive leadership, work autonomy, and status competition motivation have a three-dimensional interaction relationship with harmonious work passion.

### 5.2. Theoretical Contribution

The theoretical contributions of this paper are mainly reflected as follows:

(1) Exploring the mechanism of inclusive leadership on employees’ creative territory behavior provides a new perspective and starting point for understanding the process of inhibiting employees’ creative territory behavior. Inclusive leadership is a leadership style that understands the differentiated needs of employees and accommodates their individual characteristics, which can enhance the innovation ability and core competitiveness of the organization (Tang & Zhang, 2015). Previous studies have shown that individual factors (X. Chen et al., 2023), leadership style factors (Cao & Zhao, 2022; Huang & Tang, 2020; Tan & Yuan, 2024), interpersonal factors (Deng & Xiang, 2022), and specific organizational situations have significant effects on employee territory behavior, but the negative impact of inclusive leadership on creative territory behavior lacks in-depth theoretical construction and empirical testing.
(2) Expanding the internal mechanism between inclusive leadership and creative territory behavior by using the “environment–cognition–behavior” framework. Previous studies have shown that harmonious work passion is mainly influenced by leadership style, work characteristics, organizational environment, and individual traits (Slemp et al., 2021; Dwertmann & Boehm, 2016; Jansen et al., 2014; Nishii, 2013; Shore et al., 2011; Hao et al., 2018; Egan et al., 2019; Shore et al., 2011; D. Wang et al., 2022), and that it is conducive to stimulating employee creativity, positive organizational behavior, and cooperative willingness (G. Y. Wang et al., 2016; Vallerand et al., 2007; L. Chen et al., 2021; Ma & Ma, 2021; Vallerand et al., 2003; Lavigne et al., 2012; Yukhymenko-Lescroart & Sharma, 2019). However, few scholars analyze the mediating effect between inclusive leadership and employee creative territory behavior from the chain of “leadership behavior–psychological needs–behavioral outcomes”. Therefore, this paper examines the mediating effect of harmonious work passion on inclusive leadership and creative territory behavior, which provides a new explanation for understanding inclusive leadership to inhibit creative territory behavior by satisfying employee autonomy, competence, and relationship needs.
(3) To explore the negative effects of the interaction of inclusive leadership, work autonomy, and status competition motivation on employees’ creative territory behavior. There are many studies that agree that job characteristics and individual traits have an impact on individual behavior, but they are all analyzed from a single factor. This study introduces the dual regulation mechanism of work autonomy and status competition motivation, enriches the boundary conditions of inclusive leadership affecting employees’ creative territory behavior, and makes up for the lack of previous studies on multiple boundary conditions.

### 5.3. Practice Has Inspired

(1) Organizations should focus on fostering inclusive behavior in leadership, as well as fostering inclusive environments. Organizations should foster inclusive behavior in leaders by encouraging them to communicate openly and value employee feedback, by further integrating inclusive behavior into their performance management and incentive systems, by encouraging leaders to participate in training courses on how to behave inclusively (Offermann & Basford, 2013), and by fostering beliefs and cognitive complexity in support of diversity (Randel et al., 2018). Leaders can regularly discuss new ideas with employees, establish non-hierarchical communication channels to improve team psychological safety, and systematically promote inclusive behavior and creative culture by training to simulate inclusive leadership scenarios and establishing feedback mechanisms that encourage trial and error.
(2) Organizations should attach importance to protecting employees’ harmonious work passion. In addition to encouraging leaders to demonstrate inclusive behavior, organizations can also adopt strategies such as creating a collaborative work environment (Ho et al., 2018), empowering employees and giving positive feedback (Vallerand et al., 2003), creating a culture that supports autonomy (D. Liu et al., 2011), and activating employees’ perception of work meaning and strengthening internal drive through task diversity and goal challenge setting (Burke et al., 2015) so as to effectively stimulate employees’ harmonious work passion.
(3) Organizations should strengthen the protection of employee knowledge and creativity. The organization should take steps to clarify employee creative ownership and reduce employee creative territory behavior due to concerns about reduced competitiveness. Through intellectual property registration, project achievement recognition, and other systems, employees’ legitimate rights and interests in personal creativity are protected, and territorial defense behaviors caused by vague property rights are reduced. At the same time, employees are encouraged to actively share personal creativity, set an example of creativity sharing and experience sharing, and reward employees who contribute creativity to the organization with materials and honors.
(4) Enterprises should strengthen employees’ autonomy and guide employees’ competitive motivation correctly. In order to ensure that employees are more flexible in their work and to promote their creative problem solving, it is necessary to consider moderately enhancing work autonomy when designing work. Leaders should give employees more autonomy to stimulate innovative work results, such as managers allowing employees to work according to their own needs, as well as to determine the location and pace of full empowerment. When recruiting employees and organizing teams, we can match the work with high levels of autonomy to the employees with high levels of status competition motivation. In the process of organizational management, managers should face up to the needs of employees for status, treat the status competition among employees correctly and dialectically, and guide employees to adopt positive status competition strategies.

### 5.4. Research Limitations and Future Prospects

(1) In this study, we followed the practice of previous scholars and adopted employee self-evaluation reports. There may be a social expectation bias present that leads to the overestimation of the relationship between variables. Although we adopted multi-time point data collection to reduce instant response bias, it is still difficult to completely exclude reverse causality from cross-section data. Future studies may use multi-source evaluations (e.g., combining leadership and peer evaluations) and longitudinal follow-up designs to enhance the causal inference of conclusions.
(2) This study only discusses the moderating effect of status competition motivation and verifies the sensitivity of Chinese employees to “face”. In the future, other personality traits (such as achievement motivation and risk preference) or situational factors (such as organizational culture type and industry competition intensity) should be considered. Furthermore, this study builds a model based on self-determination theory; future studies should incorporate other theories (such as resource conservation theory and social identity theory) for cross-validation, which may increase the interpretation depth of complex mechanisms. The triple interaction effect of work autonomy and status competition motivation is not significant in some situations, which may be related to the constraints of industry or task characteristics.
(3) This study focused on individual-level analysis and did not explore aggregation effects at the team or organizational levels, such as the group impact of team inclusion climate on creative territory behavior. In reality, team interaction patterns and organizational innovation systems may strengthen or weaken individual behavior; therefore, further cross-level research at the team and individual levels may explain more complex mechanisms in the future.
(4) This study is based on a Chinese cultural context. Chinese culture may have its particularity, such as collectivism culture, which may affect the universality of the conclusions drawn. In the future, the study scenario should be expanded to verify the model in a cross-cultural context (such as comparing Chinese and Western enterprises).

## Figures and Tables

**Table 1:** Results of confirmatory factor analysis.

Model	*χ* ^2^	df	χ^2^/df	RMSEA	SRMR	CFI	TLI
Basic model: A, B, C, D	406.290	371 ***	1.095	0.021	0.040	0.992	0.991
Three-factor model: A, B, C+D	791.684	374 ***	2.117	0.070	0.096	0.906	0.898
Bi-factor model: A, B+C+D	1350.398	376 ***	3.591	0.107	0.128	0.781	0.723
One-factor model: A+B+C+D	2470.652	377 ***	6.553	0.157	0.157	0.529	0.493

Note: N = 227; *** = *p* < 0.001. A, B, C, and D represent inclusive leadership, creative territory behavior, harmonious work passion, and work autonomy, respectively.

**Table 2:** Descriptive statistics and correlation analysis.

Variable	M	SD	AVE	CR	1	2	3	4
1. Inclusive leadership	0.507	0.501	0.670	0.948	1			
2. Work autonomy	0.502	0.501	0.548	0.894	0.004	1		
3. Creative territory behavior	3.423	1.162	0.618	0.866	−0.337 **	−0.296 **	1	
4. Harmonious work passion	3.632	1.019	0.649	0.943	0.362 **	0.317 **	−0.454 **	1

Note: N = 227. Inclusive leadership and work autonomy are dummy variables. 1 = high levels of inclusive leadership group; 0 = low levels of inclusive leadership group. 1 = high levels of work autonomy group; 0 = low levels of work autonomy group. The same applies below. ** = *p* < 0.01; two-tailed test.

**Table 3:** Results of mediating effect test.

Variable	Work Autonomy			Harmonious Work Passion
	M1	M2	M3	M4
Constant	4.287 **	5.412 ***	5.425 **	2.682 **
Gender	−0.290	−0.113	−0.142	0.277 *
Age	−0.027	0.011	−0.004	0.054
Inclusive leadership	−0.787 **		−0.472 **	0.743 **
Harmonious work passion		−0.511 ***	−0.424 **	
*F*	10.792 ***	19.582 ***	17.937 ***	13.288 ***
*R* ^2^	0.127	0.547	0.244	0.152
Δ*R*^2^	0.115	0.197	0.231	0.140

Note: * means *p* < 0.05, ** means *p* < 0.01, *** means *p* < 0.001.

**Table 4:** Mediating effect values and confidence intervals of the bootstrap method at different levels of work autonomy.

Moderator Variable	Level	Indirect Mediating Effect	SE	95% Confidence Interval	
				Boot LLCI	Boot ULCI
Work autonomy	high	−0.467	0.107	−0.0.685	−0.274
	difference value	−0.304	0.120	−0.574	−0.095
	low	−0.162	0.080	−0.326	−0.009

**Table 5:** Descriptive statistical analysis results of samples.

Control Variable	Option	Frequency	%
gender	man	243	52.71
	woman	218	47.29
age	25 and younger	153	33.19
	26–30	192	41.65
	31–35	40	8.68
	36–40	58	12.58
	41 years and older	18	3.90
education background	high school and below	27	5.86
	junior college	87	18.87
	regular college course	234	50.76
	graduate student	113	24.51
position	clerk	361	78.31
	lower management	73	15.84
	middle management	22	4.77
	top management	5	1.08
tenure	1 year and below	135	29.28
	2–3	127	27.55
	4–5	112	24.30
	6–9	56	12.15
	more than 10 years	31	6.72
industry	communication	69	14.97
	IT	138	29.93
	manufacturing industry	116	25.16
	bioengineering	23	4.99
	space flight and aviation	16	3.47
	energy	69	14.97
	financial	30	6.51
total		461	100.00

**Table 6:** Results of confirmatory factor analysis.

Model	χ^2^	df	χ^2^/df	RMSEA	SRMR	CFI	TLI
Basic model: A, B, C, D, E	1328.630 ***	979	1.357	0.028	0.032	0.975	0.974
Quartet model: A, B, C, D+E	3722.482 ***	983	3.787	0.078	0.093	0.808	0.798
Three-factor model: A+B, C, D+E	4431.831 ***	986	4.495	0.087	0.114	0.758	0.746
Bi-factor model: A+B+C, D+E	5634.658 ***	988	5.703	0.101	0.131	0.674	0.659
One-factor model: A+B+C+D+E	7702.577 ***	989	7.788	0.121	0.138	0.529	0.507

Note: N = 461; *** = *p* < 0.001. A, B, C, D, and E represent inclusive leadership, creative territory behavior, harmonious work passion, work autonomy, and status competition motivation, respectively.

**Table 7:** Descriptive statistical analysis.

Variable	*M*	*SD*	*AVE*	*CR*	1	2	3	4	5	6	7	8	9	10	11
1. gender	1.47	0.50	-	-	1										
2. age	2.12	1.12	-	-	0.062	1									
3. education background	2.94	0.82	-	-	0.012	0.091	1								
4. position	1.29	0.60	-	-	0.011	0.755 **	0.009	1							
5. tenure	2.39	1.21	-	-	0.114 *	0.820 **	−0.191 **	0.633 **	1						
6. industry	3.23	1.85	-	-	−0.052	−0.017	0.044	0.011	−0.090	1					
7. A	3.72	1.00	0.606	0.933	0.016	−0.102 *	0.124 **	−0.043	−0.110 *	0.055	1				
8. B	3.65	1.12	0.639	0.876	0.020	0.178 **	−0.080	0.101 *	0.207 **	0.001	−0.494 **	1			
9. C	3.55	1.01	0.553	0.896	0.003	−0.004	0.078	0.013	−0.065	0.062	0.382 **	−0.451 **	1		
10. D	3.41	1.08	0.658	0.945	−0.009	−0.026	0.071	0.020	−0.093 *	0.086	0.322 **	−0.500 **	0.446 **	1	
11. E	3.78	0.94	0.568	0.957	−0.007	−0.055	0.047	−0.022	−0.062	0.041	0.298 **	−0.461 **	0.420 **	0.464 **	1

Note: * means *p* < 0.05, ** means *p* < 0.01. A, B, C, D, and E represent inclusive leadership, creative territory behavior, harmonious work passion, work autonomy, and status competition motivation, respectively.

**Table 8:** Results of mediating effect test.

Variable	Creative Territory Behavior			Harmonious Work Passion	
	M5	M6	M7	M8	M9
gender	−0.002	0.010	0.012	0.017	0.008
age	0.136	0.029	0.081	0.083	0.168
education background	−0.065	0.006	0.001	0.039	−0.018
position	−0.092	−0.046	−0.043	0.047	0.010
tenure	0.143	0.163 *	0.111	−0.153	−0.169
industry	0.019	0.043	0.052	0.049	0.030
inclusive leadership		−0.478 ***	−0.361 ***		0.381 ***
harmonious work passion			−0.308 ***		
F	3.956 ***	23.999 ***	30.529 ***	1.267	12.005 ***
R^2^	0.050	0.271	0.351	0.016	0.156
ΔR^2^	0.050	0.221	0.080	0.016	0.140

Note: * means *p* < 0.05, *** means *p* < 0.001.

**Table 9:** Decomposition results of total effect, direct effect, and intermediate effect.

	Effect Value	Boot SE	Boot LLCI	Boot ULCI	Relative Effect Value (Proportion of Effect)
total effect	−5.336	0.456	−6.232	−4.441	
direct effect	−4.026	0.456	−4.939	−3.112	75.45%
mediating effect of harmonious work passion	−1.311	0.2221	−1.772	−0.0909	24.57%

**Table 10:** Results of hierarchical regression of moderating effects.

Variable	Harmonious Work Passion			
	M10	M11	M12	M13
gender	0.017	0.009	0.011	0.005
age	0.083	0.144	0.151	0.144
education background	0.039	−0.015	−0.037	−0.043
position	0.047	−0.013	−0.026	−0.009
tenure	−0.153	−0.114	−0.120	−0.130
industry	0.049	0.011	−0.005	−0.005
inclusive leadership		0.232 ***	0.257 ***	0.205 ***
work autonomy		0.256 ***	0.242 ***	0.207 ***
status competition motivation		0.233 ***	0.273 ***	0.270 ***
inclusive leadership × work autonomy			0.159 ***	0.161 ***
inclusive leadership×status competition motivation			0.070	0.133 **
work autonomy × status competition motivation			0.043	0.063
inclusive leadership × work autonomy × status competition motivation				0.166 ***
*F*	10,267	22.187 ***	20.142 ***	19.858 ***
*R* ^2^	0.016	0.307	0.350	0.366
Δ*R*^2^	0.016	0.041	0.001	0.016

Note: ** means *p* < 0.01, *** means *p* < 0.001.

**Table 11:** Mediating effect values and confidence intervals of the bootstrap method at different levels of work autonomy.

Moderator Variable	Level	Indirect Mediating Effect	SE	95% Confidence Interval	
				Boot LLCI	Boot ULCI
work autonomy	high	−0.1740	0.0294	**−** **0.2342**	**−** **0.1205**
	difference value	−0.1423	0.0344	**−** **0.2126**	**−** **0.0771**
	low	−0.0317	0.0228	**−** **0.0775**	**0.0127**

Note: The boldface in the table indicates that this part is significant.

**Table 12:** Mediating effect values and confidence intervals of the bootstrap method for different moderating effects.

Work Autonomy	Status Competition Motivation	Indirect Mediating Effect	SE	95% Confidence Interval	
				Boot LLCI	Boot ULCI
2.3275	2.8393	−0.00156	0.0240	−0.0625	0.0314
2.3275	4.7210	−0.0084	0.0389	−0.0899	0.0651
4.4939	2.8393	−0.0314	0.0476	**−** **0.1158**	**−** **0.0721**
4.4939	4.7210	−0.2269	0.0390	**−** **0.3079**	**−** **0.1528**

Note: The boldface in the table indicates that this part is significant.

## Data Availability

The data supporting the findings of this study can be obtained from Shihezi University, but access to these data is subject to restrictions due to licensing agreements for the current study; therefore, they are not publicly accessible. Data are, however, available from the authors upon reasonable request and with the permission of Shihezi University.
